# MicroRNA-451 Regulates Angiogenesis in Intracerebral Hemorrhage by Targeting Macrophage Migration Inhibitory Factor

**DOI:** 10.1007/s12035-024-04207-3

**Published:** 2024-05-14

**Authors:** Shuang Bai, Ge Zhang, Shiling Chen, Xuan Wu, Jiarui Li, Jingxuan Wang, Danyang Chen, Xia Liu, Jiahui Wang, Yuanwei Li, Yingxin Tang, Zhouping Tang

**Affiliations:** Department of Neurology, Tongji Hospital, Tongji Medical College, Huazhong University of Science and Technology, Wuhan, China

**Keywords:** miR-451, MicroRNA, Intracerebral hemorrhage, Angiogenesis, Macrophage migration inhibitory factor (MIF**)**

## Abstract

Intracerebral hemorrhage (ICH) is a subtype of stroke with the highest fatality and disability rate. Up to now, commonly used first-line therapies have limited value in improving prognosis. Angiogenesis is essential to neurological recovery after ICH. Recent studies have shown that microRNA-451(miR-451) plays an important role in angiogenesis by regulating the function of vascular endothelial cells. We found miR-451 was significantly decreased in the peripheral blood of ICH patients in the acute stage. Based on the clinical findings, we conducted this study to investigate the potential regulatory effect of miR-451 on angiogenesis after ICH. The expression of miR-451 in ICH mouse model and in a hemin toxicity model of human brain microvascular endothelial cells (hBMECs) was decreased the same as in ICH patients. MiR-451 negatively regulated the proliferation, migration, and tube formation of hBMECs in *vitro*. MiR-451 negatively regulated the microvessel density in the perihematoma tissue and affected neural functional recovery of ICH mouse model. Knockdown of miR-451 could recovered tight junction and protect the integrity of blood-brain barrier after ICH. Based on bioinformatic programs, macrophage migration inhibitory factor (MIF) was predicted to be the target gene and identified to be regulated by miR-451 inhibiting the protein translation. And p-AKT and p-ERK were verified to be downstream of MIF in angiogenesis. These results all suggest that miR-451 will be a potential target for regulating angiogenesis in ICH.

**Supplementary Information:**

The online version contains supplementary material available at 10.1007/s12035-024-04207-3.

## Introduction

Primary intracerebral hemorrhage (ICH) is a spontaneous, non-traumatic rupture of parenchymal blood vessels in the brain that leads to the accumulation of blood in the brain in the absence of any underlying structural vascular lesion. It is a subtype of stroke with the highest fatality and disability rate and affects 3.41 million people worldwide each year [1]. Moreover, ICH often occurs subsequently with infection or other severe trauma, severely aggravating the condition [2, 3]. The primary brain injury in acute ICH is mainly mediated by the mass effect of the hematoma and increased intracranial pressure. Edema, oxidative, neuroinflammation, and toxicity of blood-derived products contribute to further damage in the subacute phase [4, 5]. Despite the considerable and persistent burden of ICH, there are still few effective treatments compared to ischemic stroke [6].

In the intact brain, blood vessels serve as an important vehicle to provide nutrients and carry away metabolic waste to maintain brain environmental homeostasis. In a healthy adult brain, the structure of the vascular system is in a long-term stable status [7]. Whereas, under the pathological status, angiogenesis is reactivated, and involved in disease processes [8]. In ICH, angiogenesis is an important endogenous mechanism to repair brain nerve function, and the new micro-vessel network has strong permeability, which can significantly improve the blood flow of perihematomal region, and take away the metabolites to promote hematoma removal [9–11]. In our previous studies, the three-dimensional vascular networks were reconstructed and observed by MOST (micro-optical sectioning tomography) technology and we found that new blood vessels appeared scattered in the hematoma clot in 21 days [9]. Angiogenesis has been well studied in a wide variety of central nervous system diseases, such as ischemic stroke, intracranial aneurysms, and brain tumors [8, 12–14]. Compared with ischemic stroke, there are few studies on angiogenesis after ICH, and the exact mechanism of angiogenesis after ICH remains to be discussed.

As non-coding RNAs, microRNAs (miRNAs) are 18–25 nucleotides in length and can directly cleave the target messenger RNA (mRNA) or inhibit protein synthesis associated with ribosomes through binding to their 3′-untranslated region (3′-UTR) or open reading frame region [15, 16]. The role of microRNAs has been well documented, but studying their role in stroke recovery has been minimal [17]. Cheng et al. found that 29 miRNAs (7 up, 22 down), 250 target mRNAs (136 up, 114 down), and seven small nucleolar RNA changed expression in the whole blood of ICH patients after ICH [18]. It has been reported that the regulatory mechanism of miRNA after ICH mainly involves neuronal cell death or apoptosis, neuroinflammation, oxidative stress, blood–brain barrier function, brain edema, angiogenesis, etc. [19, 20].

MiR-451 is placed on chromosome 17 at 17q11.2 which mature sequences contain 22 nucleotides in length and are highly conserved pending evolvement [21]. Recent studies have shown that miR-451 plays an important role in angiogenesis by regulating the function of vascular endothelial cells. Liu et al. discussed the role of miR-451 in the angiogenesis of osteosarcoma tumors and found that the expression of miR-451 in osteosarcoma tissues was lower than that in peritumoral normal tissues, while overexpression of miR-451 could significantly inhibit the growth and metastasis of tumor cells and angiogenesis in tumor tissues [22]. Another study showed that miR-451 is one of the most significantly downregulated miRNAs in the serum of patients with ischemic stroke, and the downregulation of miR-451 can enhance the angiogenesis ability of human umbilical vein endothelial cells (HUVECs) treated with hypoxia [23]. However, the expression changes of miR-451 after ICH and its effect on angiogenesis remain to be discussed.

In this study, we further investigated the therapeutic role of miR-451 in ICH. We found that the level of miR-451 was decreased after ICH in patients and mouse models. MiR-451 inhibited angiogenesis and neurological recovery after ICH by negatively regulating macrophage migration inhibitory factor (MIF). MiR-451 could complementarily bind to the 3′-UTR of MIF mRNA, affecting its protein translation and thus downregulating the level of MIF protein. MIF played a role in regulating angiogenesis through ERK1/2 and AKT pathways. These results suggest that miR-451 is a potential target for regulating the angiogenesis after ICH.

## Methods

### Subject and Clinical Sample Collection

This study was approved by the Ethics Committee of the Tongji Hospital, Tongji Medical College, Huazhong University of Science and Technology, China (No. TJ-IRB20211270). All participants provided written informed consent for the study.

Patients (aged 18–80 years old) admitted to the neurologic department of the Tongji Hospital from September 1st, 2023, to December 30th, 2023, for non-surgical management of acute spontaneous ICH in basal ganglion (ICH volume < 30 mL) confirmed on computed tomography (CT) scan were screened and included in ICH group. The National Institute of Health Stroke Scale (NIHSS) and Glasgow Coma Scale (GCS) scores were evaluated on days 1, 3, and 7 after onset.

Healthy subjects who underwent physical examination in the Tongji Hospital during the same period were included in the control group. Patient’s serum samples were collected on days 1, 3, and 7 after the onset of ICH, and all the samples were stored at -80°C.

### Animals

These experiments were approved by the Institute of Animal Care Committee (IACUC; protocol 4,000,152) of Tongji Medical College, Huazhong University of Science and Technology, China. MiR-451^−/−^ mice were created and donated by the laboratory of Dr. Eric Olson (University of Texas, Southwestern Medical Center) and are on a C57BL/6 J background. Control C57BL/6 J (wild-type, WT) mice were obtained from the same. All mice were housed, and experiments were performed in accordance with the National Institutes of Health (NIH) Guide for the Care and Use of Laboratory Animals under the protocol approved by the IACUC (Tongji Medical College, Huazhong University of Science and Technology). The grouping of the experimental animals was carried out in strict accordance with the principle of random grouping, which each animal had the same opportunity to be assigned to each experimental group or control group, thus avoiding the influence of human factors on the experiment.

### Mouse Model of ICH

The ICH model was conducted using our developed method [9]. Briefly, miR-451^−/−^ or miR-451^+/+^ mice (20–25 g, 10–12 weeks old, male) were placed in a stereotaxic frame using modified ear-bars fitted with blunt rubber ends designed for mice in the prone position, after intraperitoneal injection of 1% sodium pentobarbital (0.70 mg/g; Sinopharm Chemical Reagent Co., Ltd., Shanghai, China). A midline scalp incision was made, and a hole was drilled in the right skull (0.5 mm posterior and 2.0 mm lateral to bregma). A 10-μL gauge needle was attached to the micro-syringe pump and injected vertically into the right corpus striatum (3.5 mm ventral to cortical surface). Five minutes later, type VII collagenase (Sigma, St. Louis, MO, USA; 0.1 U/μL in PBS; 1 μL) was injected over a 5-min period at a constant pace (0.2 μL/min) and the needle remained at the injection site for an additional 15 min. For sham operation group, 1 μL PBS was injected in the same way. After slowly withdrawing the needle, the incision was closed, and animals were allowed to recover in a warm, non-stimulating environment with free access to food and water.

### Cell Culture and Hemin Toxicity Model In Vitro

Human brain microvascular endothelial cells (hBMECs) were cultured in DMEM high glucose medium supplemented with 10% fetal bovine serum (FBS) (Gibco, Grand Island, NY, USA) at 37°C in a 95% O_2_ and 5% CO_2_ incubator, with the medium refreshed every 3 days. Cell passages were performed when the cell density reached 80–90% confluency. hBMECs (1–5 passages, > 95% purity based on expression of CD31 (1:100, #77,699, Cell Signaling Technology, Danvers, MA, USA), a typical endothelial cell marker) were grown to 85–95% confluency before use. Cell nuclei were stained with 4,6-diamidino-2-phenylindole (DAPI, 10 μg/mL, Thermo Fisher Scientific, Waltham, MA, USA).

To mimic ICH-like conditions in vitro, hBMEC cultures were exposed to hemin (Sigma-Aldrich, St. Louis, MO, USA) for a fixed time. Briefly, hBMECs were seeded in 6-well plates at a cell density of 2 × 10^5^/mL. When the cell density reached 40–50% confluency, hemin was added into the basal medium with different concentrations (5, 10, 20, 30, 40, 50, 60, 70 μM) and cultured for 24 h. Control hBMECs were not exposed to hemin. Cell viability was assessed via a Cell Counting Kit-8 (CCK-8) assay approach.

### Specimen Preparation

Mice were euthanized with 5% isoflurane and sacrificed at corresponding timepoints post-operation by cervical dislocation. For western blot and quantitative real-time polymerase chain reaction (qPCR), brains were removed and the tissue in striatum adjacent to the hematoma or the corresponding part in sham group (about 60 mg) was isolated and stored at -80°C until processing.

For immunohistochemistry and immunofluorescence, mice were transcardially perfused with 0.9% saline, followed by 250 mL ice-cold 4% paraformaldehyde (PFA; Sinopharm Chemical Reagent Co., Ltd., Shanghai, China) in 0.1 M phosphate buffer (PBS, pH = 7.4) after anesthetized with chloral hydrate. The fixed brains were removed and post-fixed in 4% PFA for 24 h (4°C) and then gradually dehydrated in 20% and 30% sucrose in PBS (pH = 7.4, 4°C). Coronal slices of 30 μm were performed at -20°C using a constant temperature freezing microtome (CM 1860, Leica, Wetzlar, Germany). They were then gently transferred to the microslides and stored at -80°C until processing.

### RNA Extraction and Quantitative Real-Time Polymerase Chain Reaction (qPCR) Analysis

Total RNA was isolated from cells or brain tissue using TRIzol Reagent (Tiangen, Beijing, China). Total RNA from human serum was isolated using miRcute miRNA isolation kit (Tiangen, Beijing, China) following the instructions. The integrity of total RNA was detected using agarose gel electrophoresis, and the purity and concentration were detected using a spectrophotometer (BioTek synergy2, Winooski, Vermont, USA).

For miR-451 detection, 2 μg RNA was reverse transcribed by using FastKing-RT SuperMix (Tiangen, Beijing, China) at 42°C for 60 min, and 95°C for 3 min according to the manufacturer’s instructions. qPCR was performed using synthetic primers and SYBR Premix PCR kit (1 μL of 1:4 cDNA dilution was used; Tiangen, Beijing, China) in Step one plus PCR System (Applied Biosystems, Carlsbad, CA, USA). After incubation at 95°C for 15 min, samples were subjected to 40 cycles of 94°C for the 20 s and 60°C for 34 s. U6 was used as an internal reference.

For MIF detection, 1 μg RNA was synthesized using FastKing-RT SuperMix (Tiangen, Beijing, China) at 42°C for 15 min, and 95°C for 3 min according to the manufacturer’s instructions. qPCR was performed using synthetic primers and SYBR Premix PCR kit (1 μL of 1:4 cDNA dilution was used; Tiangen, Beijing, China) in Step one plus PCR System (Applied Biosystems, Carlsbad, CA, USA). After incubation at 95°C for 10 min), samples were subjected to 40 cycles of 95°C for 10 s and 60°C for 32 s. β-Actin was used as an internal reference.

The expression of target genes was analyzed according to the 2^−ΔΔCT^ method. Each reaction was performed in triplicate. Related primers were listed in Table 1.

**Table 1:** Related primers of target genes in quantitative PCR

Gene	Forward primer	Reverse primer
miR-451	5′-TGGAAACCGTTACCATTACTGAGTT-3′	
Human U6	5′-CGCTTCGGCAGCACATATAC-3′	
Mouse U6	5′-CGCTTCGGCAGCACATATAC-3′	
Human MIF	5′-CTTTGTACCGTCCTCCGGTC-3′	5′-CGTTCGTGCCGCTAAAAGTC-3′
Mouse MIF	5′-CGCTTCGGCAGCA CATATAC-3′	5′-CACGAATTTGCGTGTCATCC-3′
Human β-actin	5′-TGGAATCCTGTGGCATCCATGA-3′	5′-AATGCCTGGGTACATGGTGGTA-3′
Mouse β-actin	5′-CACTGTCGAGTCGCGTCC-3′	5′-CCTTCTGACCCATTCCCACC-3′
Mouse ZO-1	5′-GCCGCTAAGAGCACAGCAA-3′	5′-TCCCCACTCTGAAAATGAGGA-3′
Mouse Claudin 5	5′-GCAAGGTGTATGAATCTGTGCT-3′	5′-GTCAAGGTAACAAAGAGTGCCA-3′
Mouse Occludin	5′-TTGAAAGTCCACCTCCTTACAGA-3′	5′-CCGGATAAAAAGAGTACGCTGG-3′

### Protein Extraction and Western Blot Analysis

The protein of brain tissue and primary hBMECs was obtained for Western blot analysis as previously described [9]. Brain tissue or hBMECs was homogenized or lysed in ice with RIPA buffer solution (Beyotime, Shanghai, China) supplemented with phenylmethylsulfonyl fluoride (PMSF) (1:100, Sigma, St. Louis, MO, USA), phosphatase, and proteinase inhibitors (1:100, both Cell Signaling Technology, Danvers, MA, USA) for protein extraction. Protein concentration was measured by using bovine serum albumin (BCA Protein Assay Kit, Beyotime, Shanghai, China).

Each sample was resolved in 10% volume of loading buffer and was incubated for 10 min at 95°C. Processed protein samples were loaded on 15% sodium dodecylsulfate-polyacrylamide gel electrophoresis (SDS-PAGE) for electrophoresis separation and then transferred onto polyvinylidene fluoride (PVDF) membranes (GE WHATMAN, Maidstone, UK). The membranes were incubated with 5% non-fat dried milk in Tris-buffered saline (TBS) for 1 h at room temperature for blocking. Then, the membranes were incubated with primary antibodies at 4℃ for 16 h. The blots were probed with specific primary antibodies: rabbit polyclonal to MIF (1:4000, ab175189, Abcam, Cambridge, MA, USA), ERK1/2 (1:1000, #4695, Cell Signaling Technology), p-ERK1/2 (1:2000, #4370, Cell Signaling Technology, Danvers, MA, USA), AKT (1:1000, #4691, Cell Signaling Technology, Danvers, MA, USA), p-AKT (1:2000, #4060, Cell Signaling Technology, Danvers, MA, USA), and β-actin (1:1000, #4970, Cell Signaling Technology, Danvers, MA, USA). Next, membranes were washed with TBS containing 0.05% Tween 20 and then incubated with anti-rabbit immunoglobulin G (IgG) secondary antibody (1:10,000, #5151, Cell Signaling Technology, Danvers, MA, USA) for 2 h at room temperature. Membrane development was performed by an enhanced chemiluminescence-based detection method (ECL Prime Western Blotting Detection Reagent, Beyotime, Shanghai, China) using ChemiDoc MP system (Bio-Rad, Hercules, CA, USA). Finally, the intensity of blots was semi-quantified by the Image J software (National Institutes of Health, Bethesda, MD, USA). β-Actin was used as the internal control. Experiments were carried out in triplicate.

### Cell Transfection

Cell transfection was carried out using the methods described by Wan et al. [24]. Briefly, when hBMECs seeded in 6-well plates had grown to a density of 70–80%, the cells were transfected with 30 nM miR-451 mimics/negative control (NC) mimics and 100 nM miR-451 inhibitor/NC inhibitor using Lipofectamine 3000 (Invitrogen, Garlsbad, CA, USA), according to the manufacturer’s instructions. MiR-451 mimics/NC mimics and miR-451 inhibitor/NC inhibitor were designed and provided by Rebo Biotechnology Co., Ltd., (Guangzhou, China). Lipofectamine 3000 (3.75 μL) was diluted with 125 μL Opti-MEM. miR-451 mimics/NC mimics (3 μL) or miR-451 inhibitor/NC inhibitor (10 μL) were diluted with 125 μL Opti-MEM. The above two dilutions were then mixed together and incubated at room temperature for 15 min. The mixtures were added in each well with 1.75 mL Opti-MEM to reach the final concentration of 30 nM for miR-451 mimics/NC mimics and 100 nM for miR-451 inhibitor/NC inhibitor. The plates were placed in a 37°C incubator supplemented with 5% CO_2_ for 24 h. After 24 h of transfection and culturing in 37°C incubator supplemented with 5% CO_2_, total RNA was extracted from the cells with different treatments, and the level of miR-451 was measured using qPCR to evaluate the cell transfection efficiency. The miR-451sequences are shown in Table 1.

The sequences of miR-451 mimic, miR-451 inhibitor, and miR-451 agomir are shown in Supplementary Table 1.

### Tube Formation Assay

Ninety-six-well plates were pre-chilled (-20°C) and coated with 50 μL matrigel (BD Biosciences, San Jose, CA, USA) and placed at room temperature for 30 min. The hBMECs under different conditions were seeded on the surface of the Matrigel at 1.5 × 10^5^ cells/well and incubated in DMEM/HIGH GLUCOSE medium at 37°C for 24 h. A microscope (Olympus CKX31CF, Tokyo, Japan) at 100 × magnification was used to visualize the images for each well, and tube-like structures were observed and photographed from five randomly selected microscopic fields. The Image J software was used to calculate the total number of branches and tubule lengths.

### Transwell Assay

hBMECs in 200 μL DMEM/HIGH GLUCOSE (2 × 10^5^/mL) were seeded into the upper chambers of a 24-well transwell system (pore size 8 μm, Corning, Corning, NY, USA), and 800 μL basal medium was added to the lower chambers. After incubation at 37°C for 12 h, cells on the surface of filter were fixed with 4% formaldehyde and stained with 0.1% crystal violet (Beyotime, Shanghai, China). Cells were photographed and counted in five randomly chosen microscopic fields under a light microscope at 200 × magnification (Olympus CKX31CF, Tokyo, Japan).

### Wound Healing Assay

hBMECs (3 × 10^5^/mL, 2 mL) were seeded into the 6-well plates and cultured in a normal medium for 24 h at 37°C. Then, the cell monolayer was scraped by a 200-μL pipet tip following the straight lines at the outer bottom of the plates. The exfoliated cells were washed with PBS. After that, the hBMECs were cultured with fresh serum-free DMEM for 24 h. The healing of the cells was observed by a microscope (Olympus CKX31CF, Tokyo, Japan) at 100 × magnification at 0 h and 24 h. The wound healing was evaluated by measuring the width of scratches by the Image J software.

### Stereotaxic Injection of miR-451 Antagomir

To up-regulation the miR-451 level in ICH mouse model, miR-451 agomir, and NC agomir (Ribo Biotechnology Co., Ltd., Guangzhou, China) were dissolved in RNase-free ddH_2_O to reach the final concentration of 0.2 nmol/μL. Before induction of ICH, a 10-μL gauge needle was attached to the micro-syringe pump and stereotaxically advanced vertically into the right corpus striatum (3.5 mm ventral to cortical surface). Five minutes later, miR-451 and NC agomir (0.2 nmol/μL in ddH_2_O; 5 μL) was injected over a 5-min period at a constant pace (0.2 μL/min) and the needle remained at the injection site for an additional 15 min. Then, type VII collagenase 0.1 U was injected as described before. The rest of the steps were the same as above. The ipsilateral hemicerebrum was collected for detection on seventh day after administration.

### Behavioral Tests

Behavioral tests, including modified neurological severity scores (mNSS), corner test, and forelimb placing test, were carried out on days 0, 1, 3, 5, 7, 14, and 21 after surgery.

To assess the neurological deficits of mice in terms of motor, sensory, balance, reflex, and abnormal movements, mNSS was employed [25]. Each mouse was tested five times. A higher score indicates a more severe injury.

The corner test was used to detect the integrity of motor and sensory [26]. Mouse was allowed to proceed into a corner, the angle of which was 30°. To exit the corner, the mouse could turn either to the left or the right, and this was recorded. This was repeated 10 times per mouse, and the percentage of right turns was calculated.

For the vibrissae-elicited forelimb placing test, mice were held by their torsos, which allowed the forelimb to hang free. Independent testing of each forelimb was induced by brushing the respective vibrissae on the corner edge of a countertop. Intact animals place the forelimb ipsilateral to the stimulated vibrissae quickly onto the countertop, which may be impaired in ICH mice depending on the extent of injury. In the experiments, each mouse was tested 10 times for each forelimb, and the percentage of trials in which the mouse placed the appropriate forelimb on the edge of the countertop in response to the vibrissae stimulation was determined [26].

Testers were highly experienced and blind to the condition of the animal.

### Measurement of Vessel Density by Immunofluorescent Staining

For immunofluorescent staining, brain slices were immersed in the EDTA-antigen retrieval solution (PH 8.0, BioGenex, Fremont, California, USA) for 4 min with intermittent microwave irradiation (4 s on/3 s off at 200 W). Permeabilize and block the tissue with 0.5%Triton X-100 in PBS with 3% BSA at room temperature for 1 h. Rinse specimens with PBS several times; incubation then was performed with one of the primary antibodies: rabbit anti-CD31 (1:50, Cell Signaling Technology, Danvers, MA, USA) at 4°C for 16 h. Rinse specimens with PBS several times; brain slices were incubated with Cy3-labeled secondary antibody (goat anti-rabbit IgG, 1:100, Thermo Fisher Scientific, Waltham, MA, USA) at room temperature for 1 h, away from light. Images were acquired by confocal microscopy (Olympus BX53, Tokyo, Japan). Three visual fields at 100 × magnification around the hematoma from three brain slices of each mouse were selected for statistical analysis. Microvessel density was evaluated by a number of microvessels in each field. Image analysis was performed with the Image J software. In the slices immunostained for CD-31, immunepositive cells were counted by an investigator who was blinded to different treatment groups in six fields (cells/mm^2^) in the median part at 0 to 0.5 mm around the hematoma.

### Measurement of Vessel Density by FITC-Perfused

Three weeks after modeling, mice were intracardiac perfused with PBS (pH 7.0) containing 10 mM Dextran for 5 min and fluorescein isothiocyanate-dextran (FITC-dextran 0.1 mg/mL, 40 mL/mouse; Sigma-Aldrich, St. Louis, MO, USA) in PBS (pH 7.0) for 3 min in sequence. After that, the brain tissue was fixed by intracardiac perfusion with ice-cold 4% PFA (pH 8.0) in 0.1 M PB 100 mL for 10 min. Then, the brains were removed and postfixed in 4% PFA in 0.1 M PBS (pH 8.0) for 24 h at 4°C and cryoprotected by 30% sucrose in PBS (pH 8.0) for 24 h at 4°C. Coronal slices of 30 μm were performed at -20°C using a constant temperature freezing microtome (CM 1860, Leica, Wetzlar, Germany). Images were acquired by fluorescent microscopy (Olympus BX53, Tokyo, Japan). Three visual fields around the hematoma from three brain slices of each mouse were selected for statistical analysis. Image analysis was performed with the Image J software.

### Dual Luciferase Reporter Assays

Luciferase reporter vectors encoding the complete wild type 3′ UTR of the human and mouse MIF mRNA (hMIF WT and mMIF WT), as well as parallel control vectors containing nucleotide mutations in the predicted miR-451 binding sites (hMIF MUT and mMIF MUT), were constructed by Ribo (Ribo Biotechnology Co., Ltd., Guangzhou, China). Seven nucleotide mutations of human control vectors located in the position 102–108 of hMIF 3′ UTR (117 bp), which mutated from AACGGTT to TTGCCAA. Seven nucleotide mutations of human control vectors located in the position 106–112 of mMIF 3′ UTR (124 bp), which mutated from to AATGGTT to TTACCAA. Luciferase constructs were transfected into HEK293T cells together with the (h/m)MIF-WT 3′UTR vector (0.2 μg) or the (h/m)MIF-MUT 3′UTR vector (0.2 μg) and miR-451 mimics (30 nM) or NC mimics(30 nM). Cells were cultured in 24-well plates for 48 h, and luciferase activity was detected by the dual-luciferase reporter assay system (Promega, Madison, WI, USA).

### Administration of ISO-1

ISO-1 (Selleck Chemicals, Houston, TX, USA), as a specific inhibitor of MIF, was dissolved in DMSO to the concentration of 200 mg/mL and stored at -80°C. The storage was diluted to a concentration of 4 mg/mL for use. The mice were given the dose of 35 mg/kg by intraperitoneal injections twice a day and the drug administration lasted for five days from the day of modeling.

### Statistical Analysis

Statistical analyses were performed with GraphPad Prism (Version 8.0; La Jolla, CA, USA). For data from more than two groups with normal distribution and homogeneity of variance, one-way analysis of variance (ANOVA) was used for data processing with Turkey’s Test. For the data of behavior data evaluation, Kolmogorov–Smirnov test with Dallal-Wilkinson-Lilliefor *P* value was used to test if the values come from a Gaussian distribution. Two-way ANOVA and Geisser-Geenhouse correction were used to analyze the data. Data were expressed as mean ± standard deviation (SD). The difference was considered to indicate statistical significance at *P* < 0.05.

## Results

### Expression of miR-451 in the Peripheral Blood of ICH Patients in the Acute Stage Was Decreased

**Fig. 1:**
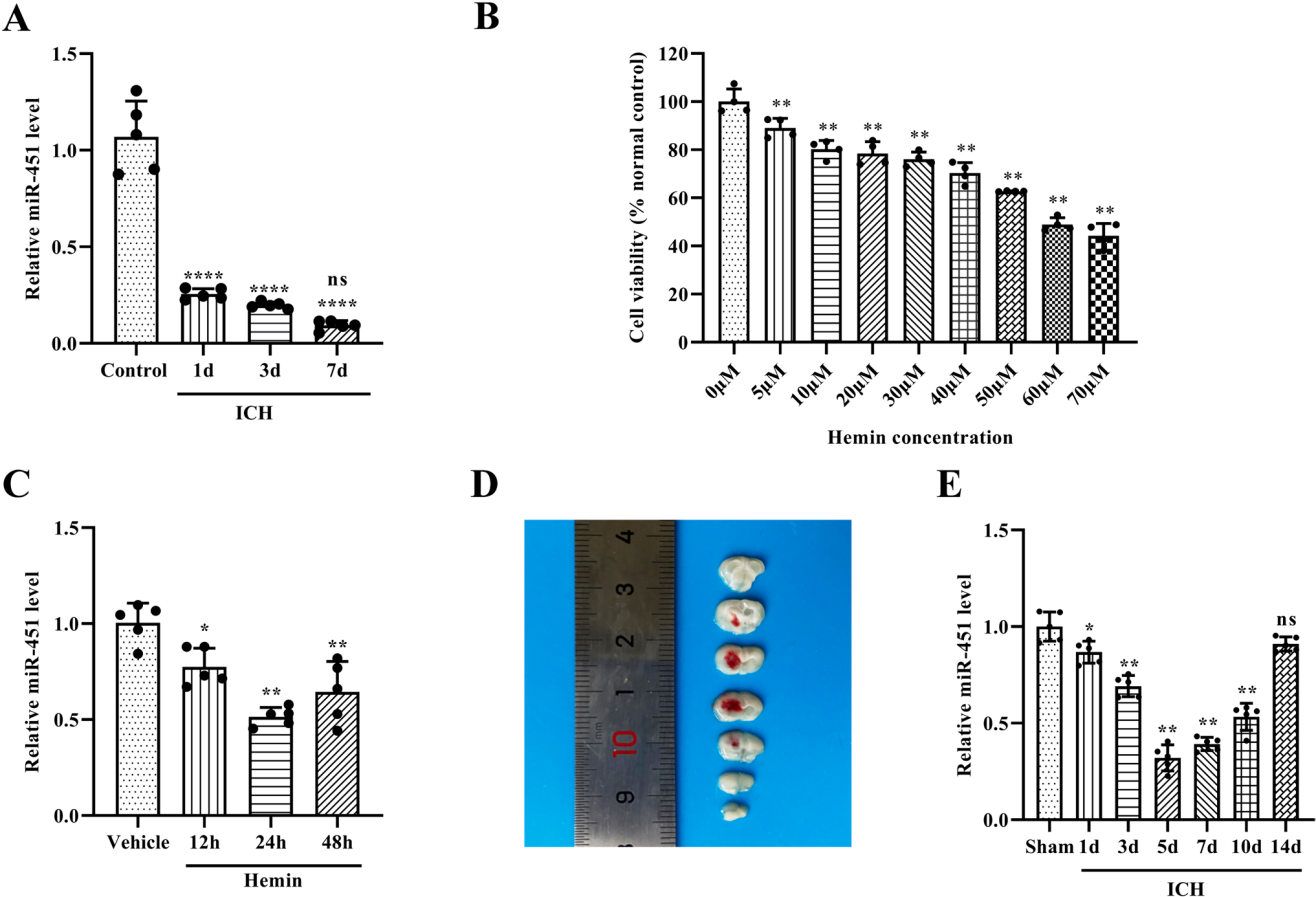
MiR-451 expression was significantly decreased after ICH. **A** Relative expression levels of miR-451 were detected by qPCR in the peripheral blood of patients on days 1, 3, and 7 after ICH. ^****^P < 0.001, versus the control group. ns, no significance, versus the day 1 group, *n* = 5; **B** CCK-8 assay detected the cell viability when hBMECs were exposed to hemin treatment with different concentrations (0, 5, 10, 20, 30, 40, 50, 60, 70 μM) for 24 h. ^**^P < 0.01, versus the 0 μM group, *n* = 4. **C** Relative expression levels of miR-451 were detected by qPCR in hBMECs exposed to hemin (60 μM) for 12 h, 24 h, and 48 h. ^*^P < 0.05, ^**^P < 0.01, versus the vehicle group, *n* = 4. **D** A round-like dark red hematoma in the right corpus striatum in mouse ICH model. **E** Relative expression levels of miR-451 were detected by qPCR in the perihematoma tissue in ICH mouse model. ^*^P < 0.05, ^**^P < 0.01, ns, no significance, versus the sham group, *n* = 4. Data are presented as means ± SD. One-way ANOVA was used to process the data, and Turkey test was performed

qPCR analysis demonstrated that the expression of miR-451 was significantly decreased in the peripheral blood of ICH patients in the acute stage (from 1st day to 7th day after ICH). Compared to healthy controls, the expression of miR-451 decreased to 23.87% on day 1, 18.48% on day 3, and 8.76% on day 7, respectively (Fig. 1A). The clinical characteristics of the ICH patients and healthy control group were described in Table 2. Information on the condition and neurologic assessment of patients with ICH was provided in the Supplementary Table 2.

**Table 2:** Clinical characteristics of the ICH patients and healthy control group who were used for analyzing the expression of miR-451

		ICH patients (*n* = 5)	Healthy control (*n* = 5)	*P* value
Age (years, mean ± SD)		65.20 ± 4.26	66.00 ± 5.02	0.8141
Sex, male (%)		60%	60%	1
BP (mmHg, mean ± SD)	SBP	179.6 ± 31.44	127.6 ± 9.85	0.0135
	DBP	87.0 ± 7.67	73.0 ± 8.79	0.0431
Height (m, mean ± SD)		1.61 ± 0.05	1.62 ± 0.11	0.9225
Weight (kg, mean ± SD)		61.80 ± 6.71	63.62 ± 8.73	0.7495
BMI (kg/m^2^, mean ± SD)		23.96 ± 3.54	24.32 ± 1.31	0.8534

*BP* blood pressure, *SBP* systolic blood pressure, *DBP* diastolic blood pressure, *BMI* body mass index, *SD* standard deviation

### Expression of miR-451 in the ICH Mouse Model and Hemin Toxicity Model In Vitro Was Decreased

After finding the association between ICH in humans and miR-451 reduction, we concluded that miR-451 might have therapeutic potential for ICH. To clarify the causal relationship between miR-451 reduction and ICH and the therapeutic effect of miR-451, we further conducted experiments in the ICH mouse model, and in a hemin toxicity model of hBMECs. Hemin was selected to stimulate hBMECs to construct ICH model in vitro. To find the optimal concentration of hemin for research, hBMEC cultures were exposed to hemin with different concentrations (5, 10, 20, 30, 40, 50, 60, and 70 μM) and cultured for 24 h. CCK-8 assay detected a concentration-dependent decrease of cell viability when hBMECs were exposed to hemin treatment with different concentrations. The cell viability decreased by approximately 50% at a concentration of 60 μM, which was selected as the concentration of hemin for subsequent research (Fig. 1B).

To find the optimal time of exposure to hemin, hBMEC cultures were exposed to hemin (60 μM) for 12 h, 24 h, or 48 h, respectively [27]. qPCR analysis demonstrated that the expression of miR-451 was significantly decreased and peaked at 24 h (51.22% *vs.* vehicle; *P* < 0.01; Fig. 1C). Exposure time to hemin was set as 24 h in subsequent research.

Type VII collagenase (Sigma, St. Louis, MO, USA) was injected into the right corpus striatum to simulate ICH injury in vivo (Fig. 1D). Similar to those of human blood samples, qPCR analysis confirmed the remarkable downregulation of miR-451 expression in perihematoma tissue, which dropped to its lowest point at day 5 post-ICH in ICH mouse model (32.02% *vs.* sham group; *P* < 0.01; Fig. 1E) and returned to the baseline level at day 14 post-ICH (91.01% *vs.* sham group; *P* > 0.05; Fig. 1E).

### MiR-451 Negatively Regulated the Proliferation, Migration, and Tube Formation of hBMECs in Hemin Toxicity Model In Vitro

Decreased level of miR-451 in the ICH mouse model and hemin toxicity model in vitro has been demonstrated. The influence of miR-451 on angiogenesis function of hBMECs was tested. Briefly, hBMVECs were transfected with miR-451 mimics/inhibitor and then cultured in normal or 60 μM hemin-containing medium for 24 h. The angiogenesis function of hBMECs in vitro was assessed by wound healing assay, transwell assay, and tube formation assay.

**Fig. 2:**
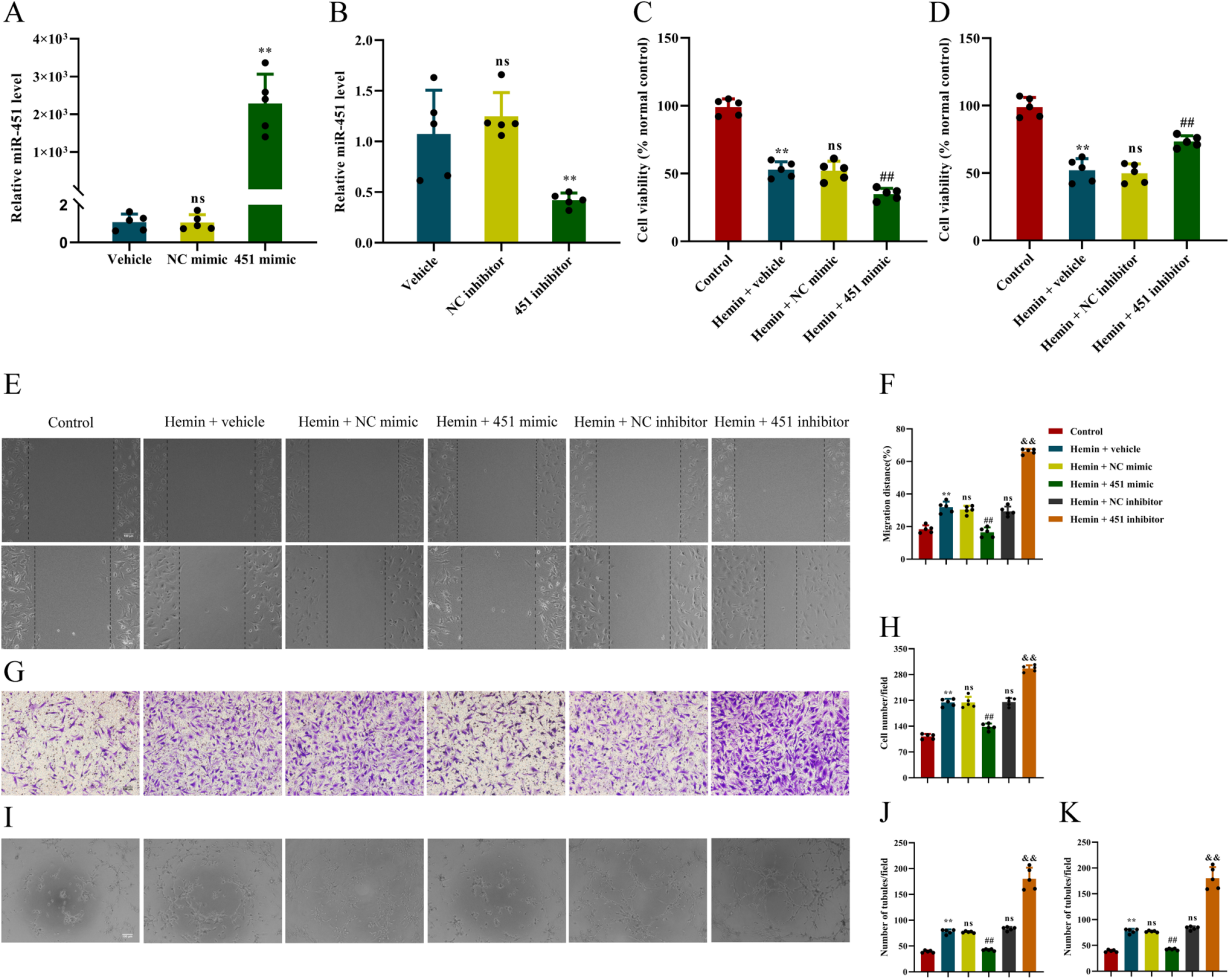
MiR-451 regulated the proliferation, migration, and tube formation of hBMECs in hemin toxicity model. **A**–**B** The expression levels of miR-451 in each group were detected by qPCR. ^**^P < 0.01, ns, no significance, versus the vehicle group, *n* = 4. **C**–**D** Cell proliferation was detected by CCK-8 assay in each group. ^**^P < 0.01, ns, no significance, versus the vehicle group, *n* = 4. **E** The wound healing assay in each treatment group was performed at 0 h and 24 h, scale = 100 μm. **F** Quantitative statistics of cell migration distance in each treatment group. ^**^P < 0.01, versus the control group. Ns, no significance, versus the hemin + vehicle group. ^##^*P* < 0.01, versus the hemin + NC mimic group. ^&&^*P* < 0.01, versus the hemin + NC inhibitor group, *n* = 5. **G** Transwell assay was performed 12 h after each treatment group. Scale = 100 μm. **H** The number of hBMECs penetrated the membrane in each treatment group was quantitatively counted. ^**^P < 0.01, versus the control group. Ns, no significance, versus the hemin + vehicle group. ^##^*P* < 0.01, versus the hemin + NC mimic group. ^&&^*P* < 0.01, versus the hemin + NC inhibitor group, *n* = 5. **I** Tube formation assay of each treatment group was performed for 24 h, scale = 100 μm. **J** The number of tubular structures formed by each treatment group was quantitatively counted, ^**^P < 0.01, versus the control group. ns, no significance, versus the hemin + vehicle group. ^##^*P* < 0.01, versus the hemin + NC mimic group. ^&&^*P* < 0.01, versus the hemin + NC inhibitor group, *n* = 5. **K** The total length of tubular structures formed by each treatment group was quantitatively counted, ^**^P < 0.01, versus the control group. ns, no significance, versus the hemin + vehicle group. ^##^*P* < 0.01, versus the hemin + NC mimic group. ^&&^*P* < 0.01, versus the hemin + NC inhibitor group, *n* = 5. Data are presented as means ± SD. One-way ANOVA was used to process the data, and Turkey test was performed

qPCR detected 2287-fold increase in expression of miR-451 in hBMECs by miR-451 mimics transfection, compared with NC mimics transfection (*P* < 0.01; Fig. 2A), and 0.39-fold decrease in expression of miR-451 in hBMECs by miR-451 inhibitor transfection, compared with NC inhibitor transfection (*P* < 0.01; Fig. 2B).

CCk-8 assay showed cell viability decreased to 66.92% by miR-451 mimic transfection, compared with NC mimic transfection (*P* < 0.05; Fig. 2C), and 1.47-fold increased by miR-451 inhibitor transfection, compared with NC inhibitor transfection (*P* < 0.01; Fig. 2D), in hemin toxicity model in vitro.

The wound healing assay detected the hBMEC migration distance increased in hemin toxicity model in vitro. The cell migration distance decreased to 45.66% by miR-451 mimics transfection, compared with NC mimic transfection (*P* < 0.05; Fig. 2E–F), and showed a 2.49-fold increase by miR-451 inhibitor transfection, compared with NC inhibitor transfection (*P* < 0.01; Fig. 2E–F), in hemin toxicity model in vitro.

Transwell assay revealed that hBMECs penetrated the membrane increased in hemin toxicity model in vitro. The number of cells penetrating the membrane decreased to 67.61% by miR-451 mimic transfection, compared with NC mimic transfection (*P* < 0.05; Fig. 2G–H), and showed a 1.44-fold increase by miR-451 inhibitor transfection, compared with NC inhibitor transfection (*P* < 0.01; Fig. 2G–H), in hemin toxicity model in vitro.

Tube formation assay revealed an increase of hBMEC tube formation in hemin toxicity model in vitro. The number of tubules and normalized total tubule length decreased to 55.06% and 54.73%, respectively, by miR-451 mimic transfection, compared with NC mimic transfection (*P* < 0.01; Fig. 2I–K), and showed a 2.16-fold and 2.21-fold increase by miR-451 inhibitor transfection, compared with NC inhibitor transfection (*P* < 0.01; Fig. 2I–K), in hemin toxicity model in vitro.

### MiR-451 Negatively Regulated the Angiogenesis in the Perihematoma Tissue of ICH Mouse Model

**Fig. 3:**
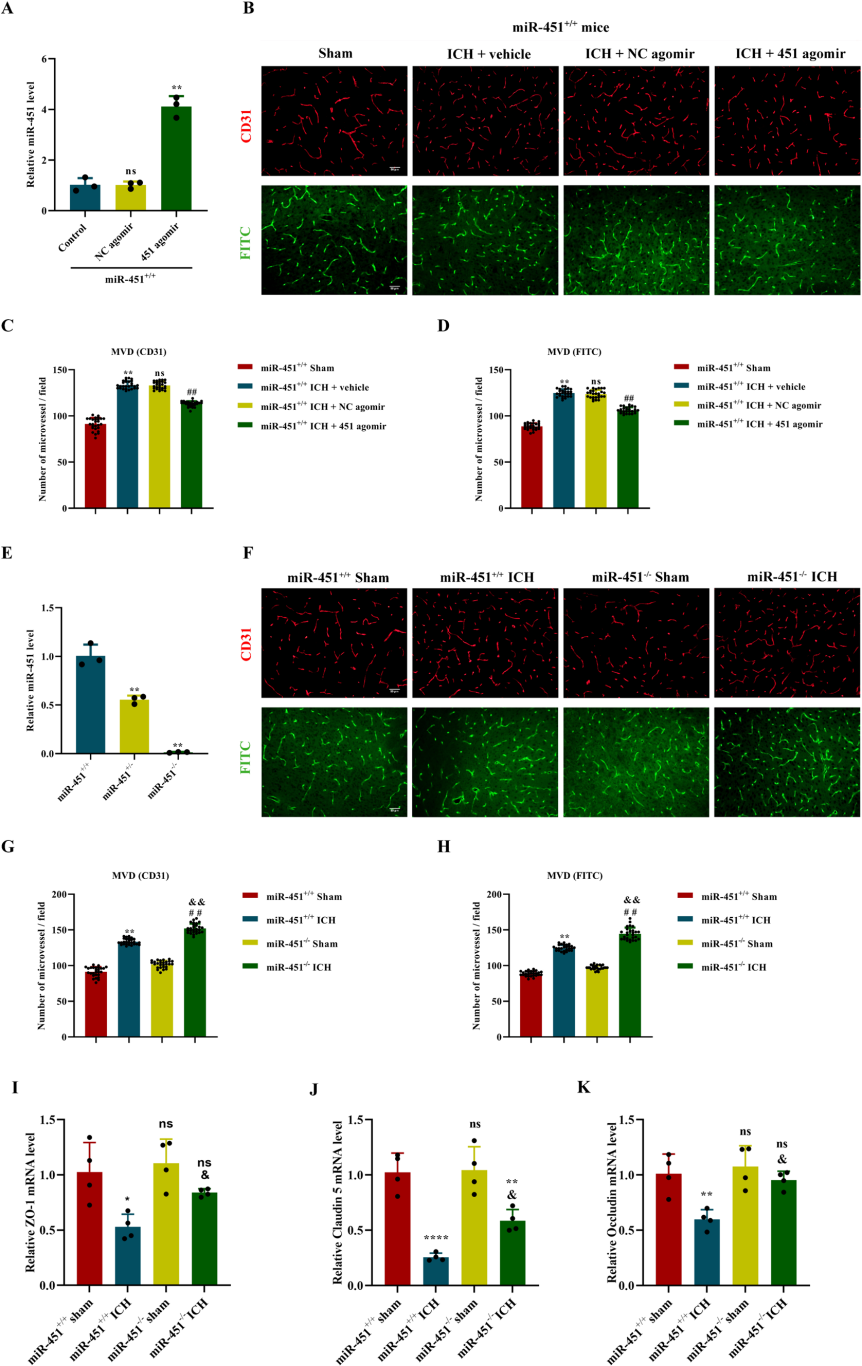
MiR-451 regulated the angiogenesis in the perihematoma tissue of the ICH mouse model. **A** The relative expression of miR-451 in the brain tissue was determined by qPCR after the injection of 451 agomir/NC agomir, ^**^P < 0.01, versus NC agomir. Ns, no significance, versus the control group, *n* = 3. **B**, **F** CD31 immunofluorescence staining of brain tissue and FITC-Dextran perfusion staining 21 days in ICH mouse model in each group, scale = 50 µm. **C**–**D** The number of blood vessels in CD31 immunofluorescence staining and FITC cardiac perfusion section was quantitatively counted; ^**^P < 0.01, versus the sham group. ns, no significance, versus the ICH + vehicle group. ^##^*P* < 0.01, versus the ICH + NC agomir group, *n* = 27. **E** The relative expression of miR-451 in the brain tissue of miR-451^+/+^ and miR-451^−/−^ mice was determined by qPCR, ^**^P < 0.01, versus miR-451^+/+^ group, *n* = 3. **G**–**H** The number of blood vessels in CD31 immunofluorescence staining and FITC cardiac perfusion section was quantitatively counted; ^**^P < 0.01, versus the miR-451^+/+^ + sham group. ^##^*P* < 0.01, versus the miR-451^−/−^ + sham group. ^&&^*P* < 0.01, versus the miR-451^+/+^ + ICH group, *n* = 27. **I**–**K** The relative expression of ZO-1, Claudin 5, and Occludin in the tissues of the perihematoma in miR-451^+/+^ and miR-451^−/−^ mice was determined by qPCR, ^*^P < 0.05, ^**^P < 0.01, ^****^P < 0.001, ns, no significance, versus THE miR-451^+/+^ + sham group. ^&^*P* < 0.05, versus THE miR-451^+/+^ + ICH group. Data are presented as means ± SD. One-way ANOVA was used to process the data, and Turkey test was performed

To find the role of miR-451 overexpression in angiogenesis in the perihematoma tissue of ICH mouse model, miR-451 agomir was injected into the right corpus striatum before ICH modeling. The expression of miR-451 in perihematoma tissue showed a 4.05-fold increase as compared with NC agomir, on the 7th day after ICH (*P* < 0.01; Fig. 3A). Microvessel density in perihematoma tissue, detected by CD31 immunofluorescent staining and FITC-Dextran perfusion staining, decreased to 84.92% and 85.89%, respectively, by miR-451 agomir injection, compared with NC agomir injection at 21st day after ICH (*P* < 0.01; Fig. 3B–D).

To investigate whether downregulate miR-451 affected angiogenesis in the perihematoma tissue, we constructed ICH model using miR-451^−/−^ mice with miR-451^+/+^ mice as the control. MiR-451 expression in brain tissue of miR-451^+/+^ and miR-451^−/−^ mice was shown in Fig. 3E. Microvessel density in perihematoma tissue, detected by CD31 immunofluorescent staining and FITC-Dextran perfusion staining, both showed a 1.49-fold increase in miR-451^−/−^ ICH mouse model, compared with miR-451^+/+^ ICH mouse model, at 21st day after ICH (*P* < 0.01; Fig. 3F–H).

The blood–brain barrier (BBB) is composed of the endothelium of cerebral capillaries, basement membrane, and perifoot of astrocytes. The tight junctions of microvascular endothelial cells are an important structural basis for maintaining the integrity of the BBB [28]. The TJ transmembrane proteins include the integral membrane proteins, occludin and claudins (for example, claudin 5, 3, 12, 1) and an IgG type of protein, junctional adhesion molecule. And zonula occludens-1 (ZO-1) establishes interactions with these transmembrane proteins [29]. To investigate whether miR-451 affected the dysfunction of BBB after ICH, qPCR was used to detect the mRNA expression levels of tight junctions proteins in each group of mice on day14 after ICH. The expression levels of ZO-1, Claudin 5, and Occludin were decreased after ICH compared to the sham group. The results showed that the tight junctions of vascular endothelial cells in mice was damaged. And the BBB dysfunction in miR-451^−/−^ ICH group was less than that in miR-451^+/+^ ICH group, indicating that knocking out miR-451 could reduce the damage of tight junction of vascular endothelial cells (*P* < 0.05; Fig. 3I–K).

### Macrophage Migration Inhibitory Factor (MIF) Was a Direct Target of miR-451

**Fig. 4:**
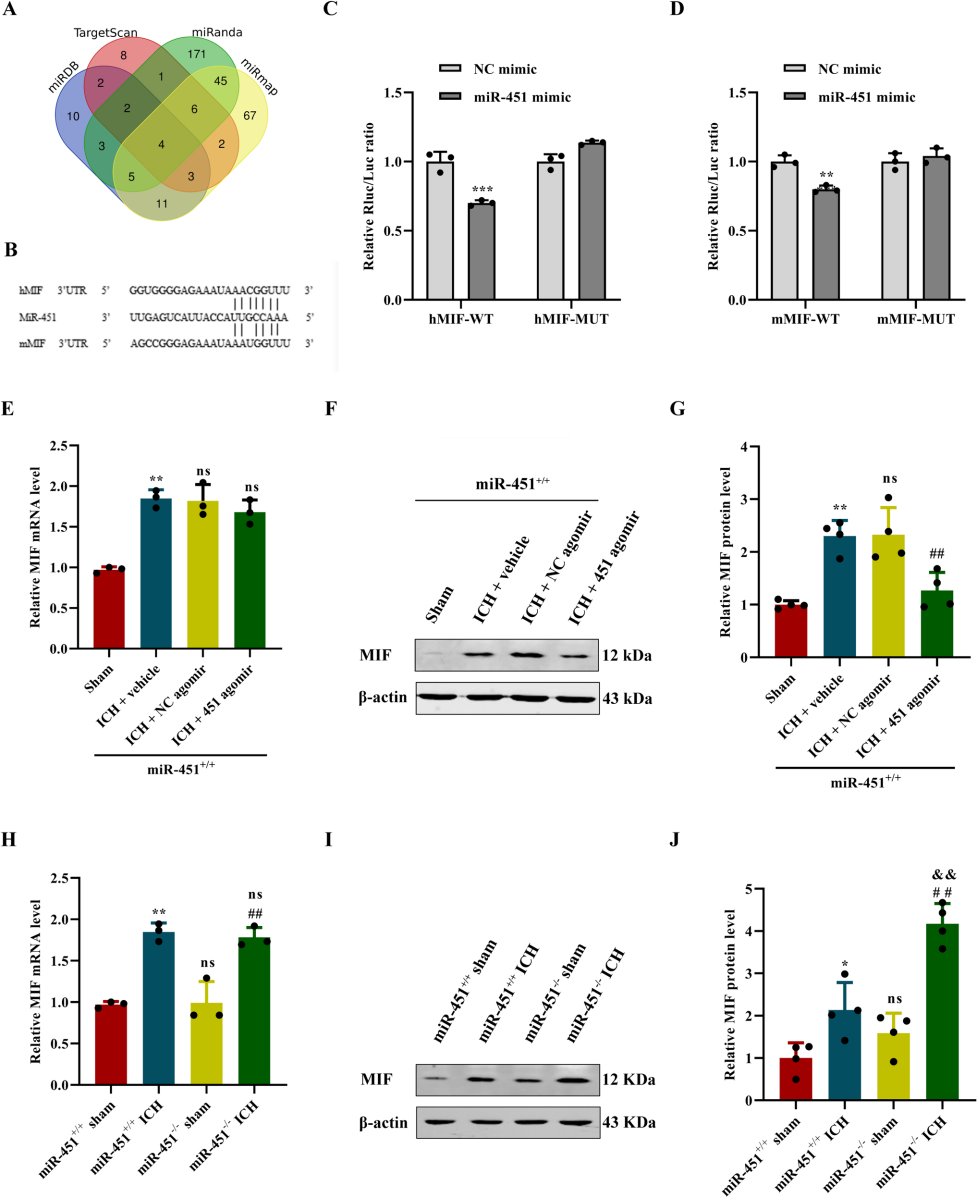
MiR-451 directly targeted MIF and negatively regulated angiogenesis after ICH. **A** Bioinformatic programs such as TargetScan, miRanda, miRmap, and miRDB were used to predict target genes. **B** Target region between human-MIF (hMIF) 3′UTR or mouse-MIF (mMIF) 3′UTR. **C** Dual luciferase report assay confirmed that hMIF was the direct target of miR-451, ^***^P < 0.01, versus hMIF-WT + miR-451 mimic, *n* = 3. **D** Dual luciferase report assay confirmed that mMIF was the direct target of miR-451, ^**^P < 0.01, versus mMIF-WT + miR-451 mimic, *n* = 3. **E** Relative expression level of MIF mRNA in the brain tissues of mice in each group was detected by qPCR. ^**^P < 0.01, ns, no significance, versus the sham group. ns, no significance, versus the ICH + NC agomir group, *n* = 4. **F**–**G** Relative expression level of MIF protein in the brain tissues of mice in each group was detected by western blot. ^**^P < 0.01, versus the sham group; ns, no significance, versus the ICH + vehicle group. ^##^*P* < 0.01, versus the ICH + NC agomir group, *n* = 4. **H** Relative expression level of MIF mRNA in the brain tissues of mice in each group was detected by qPCR. ^**^P < 0.01, versus the miR-451^+/+^ + sham group. ^##^*P* < 0.01, versus the miR-451^−/−^ + sham group; ns, no significance, versus the miR-451^+/+^ + ICH group, *n* = 4. **I**–**J** Relative expression level of MIF protein in the brain tissues of mice in each group was detected by western blot. ^**^P < 0.01, versus the miR-451^+/+^ + sham group; ns, no significance, versus the miR-451^+/+^ + sham group. ^##^*P* < 0.01, versus the miR-451^−/−^ + sham group; ^##^*P* < 0.01, versus the miR-451^−/−^ + ICH group. ^&&^*P* < 0.01, versus the miR-451^+/+^ + ICH group, *n* = 4. Data are presented as means ± SD. One-way ANOVA was used to process the data, and Turkey test was performed

Based upon bioinformatic programs such as TargetScan, miRanda, miRmap, and miRDB, which predict miRNA targets, macrophage migration inhibitory factor (MIF) is one of the four proposed targets of miR-451, with one potential conserved binding sites in its 3′untranslated region (UTR) (Fig. 4A–B). MiR-451 had seven pairs of complementary bases with human-MIF (hMIF) 3′UTR and six pairs of complementary bases with mouse-MIF (mMIF) 3′UTR (Fig. 4B).

To determine if miR-451 binds to the 3′UTR of the MIF transcript to regulate MIF translation, dual luciferase reporter assays were performed. Analysis of luciferase activity indicated that ectopic expression of miR-451 inhibited the expression of the reporter vector containing hMIF-WT 3’UTR and mMIF-WT 3′UTR but not the reporter vector containing hMIF-MUT 3′UTR and mMIF-MUT 3′UTR (Fig. 4C–D). Forced expression of the negative control mimics had no effect on luciferase activity in cells transfected with either the (h/m)MIF-WT or (h/m)MIF-MUT 3’UTR constructs demonstrating the specificity of the effect (Fig. 4C–D).

### MiR-451 Regulated the Expression of MIF Protein in ICH Mouse Model

In order to confirm the mRNA and protein levels of MIF are regulated by miR-451 after ICH in vivo, miR-451 agomir was injected into the right corpus striatum to up-regulate miR-451 level of ICH miR-451^+/+^ mouse model. On the 5th day after ICH, the mRNA and protein levels of MIF in perihematoma brain tissue both increased significantly in ICH groups as compared to the sham group (*P* < 0.05; Fig. 4E–G). mRNA levels of MIF in ICH miR-451^+/+^ mice injected with miR-451 agomir and NC agomir have no statistic difference (*P* > 0.05; Fig. 4E). However, protein level of MIF in ICH miR-451^+/+^ mice injected with miR-451 agomir decreased to 54.51%, as compared with ICH miR-451^+/+^ mice injected with NC agomir (*P* < 0.05; Fig. 4F–G).

Next, to downregulate miR-451 level, ICH mouse model was made using miR-451^−/−^ mouse. On the 5th day after ICH, the mRNA and protein levels of MIF in perihematoma brain tissue both increased significantly in both miR-451^−/−^ and miR-451^+/+^ mice after ICH as compared to the sham group (*P* < 0.05; Fig. 4H–I). miR-451^−/−^ mice showed a considerable increase in MIF protein expressions compared to miR-451^+/+^ mice after ICH (*P* < 0.05; Fig. 4I–J), but no statistical difference was detected in MIF mRNA level (Fig. 4H).

### MiR-451 Regulated Angiogenesis by Targeting MIF

**Fig. 5:**
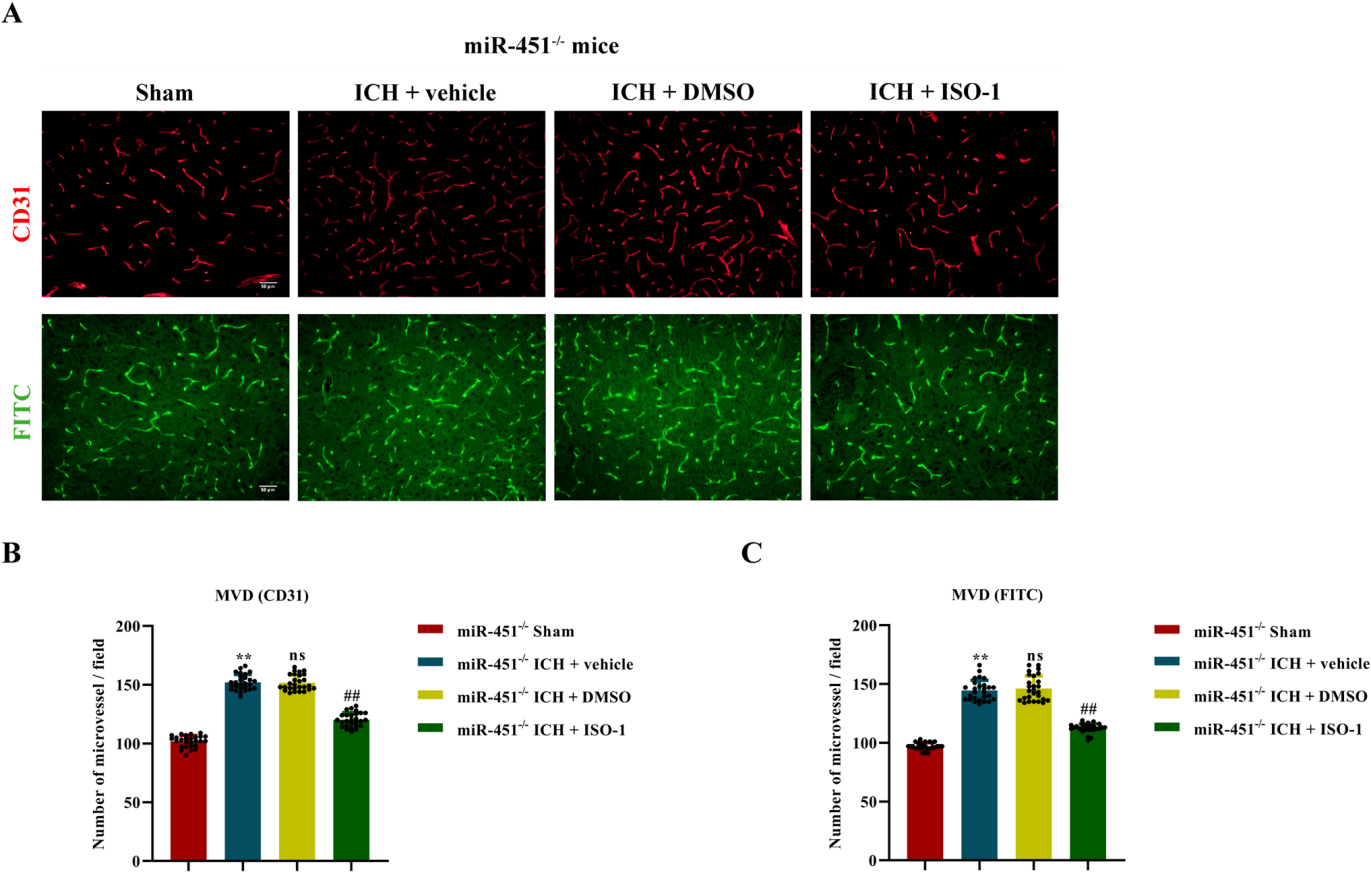
Angiogenesis in brain tissue after ISO-1 (the MIF inhibitor) treatment. **A** CD31 immunofluorescence staining of brain tissue and FITC-Dextran perfusion staining 21 days in ICH mouse model in each group, scale = 50 µm. **B**–**C** The number of blood vessels in CD31 immunofluorescence staining and FITC cardiac perfusion section was quantitatively counted. ^**^P < 0.01, versus the sham group. Ns, no significance, versus the ICH + vehicle group. ^##^*P* < 0.01, versus the ICH + DMSO group, *n* = 27. Data are presented as means ± SD. One-way ANOVA was used to process the data, and Turkey test was performed

It has been found that miR-451 directly targeted MIF, and we further investigated whether miR-451 regulated angiogenesis by targeting MIF. ISO-1, the MIF inhibitor, was injected by intraperitoneal (35 mg/kg, 2 times a day, for 5 days) after ICH modeling in miR-451^−/−^ mice. CD31 immunofluorescent staining and FITC-Dextran perfusion staining showed that microvessel density in perihematoma tissue decreased to 79.27% and 76.86% in mice injected with ISO-1 as compared with NC group on the 21st day after ICH (*P* < 0.01; Fig. 5A–C). It was confirmed that the suppression effect of miR-451 on angiogenesis was attenuated by knocking down MIF. In a word, miR-451 regulated angiogenesis by targeting MIF.

### MiR-451 Regulated MIF Expression via p-AKT and p-ERK Pathway

**Fig. 6:**
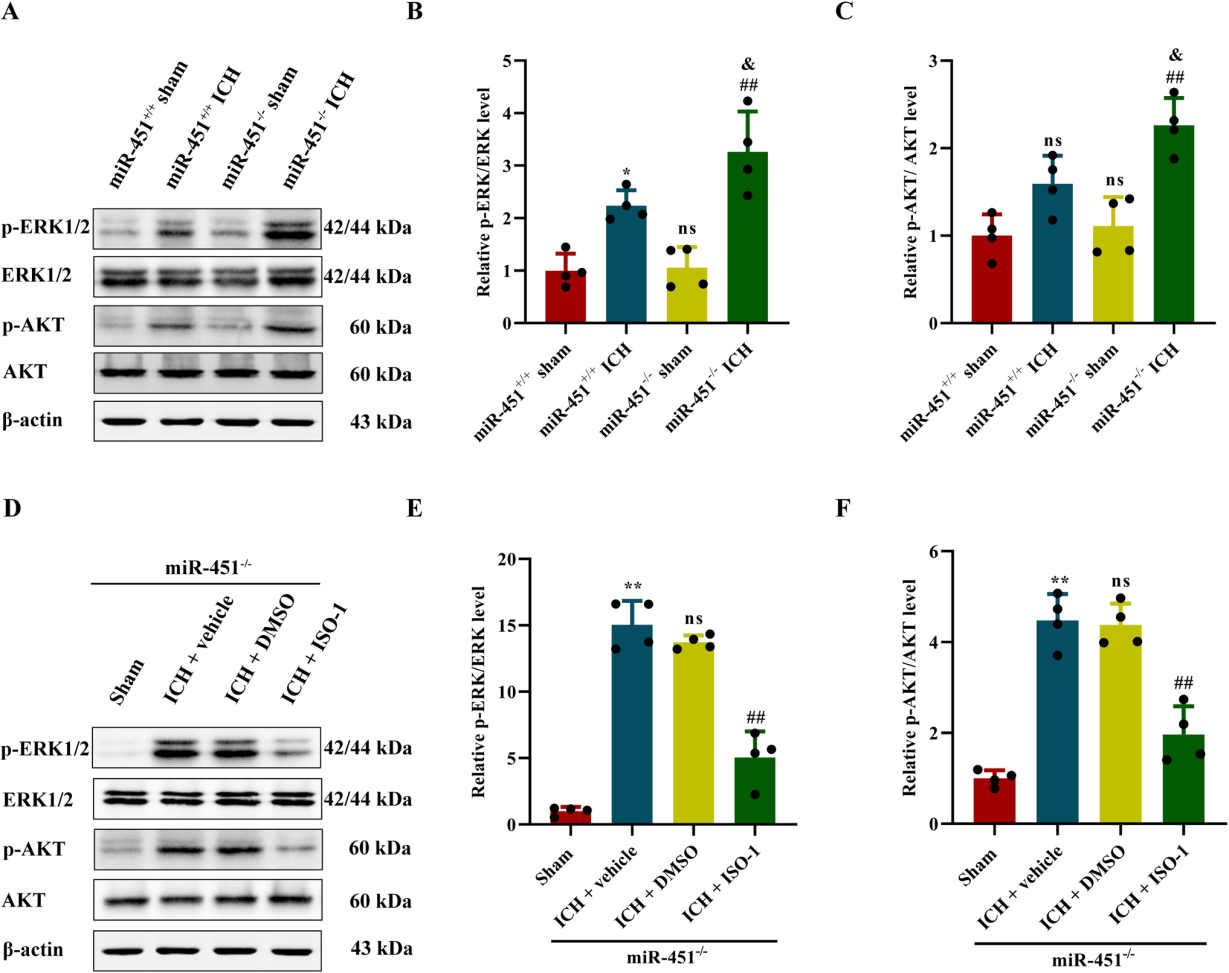
ISO-1 alleviated the effect of miR-451 knockout and regulated the ERK1/2 and AKT pathway. **A**–**C** The relative expression level of p-ERK1/2, ERK1/2, p-AKT, and AKT protein in the brain tissues of mice in each group was detected by western blot, ^*^P < 0.05, versus the miR-451^+/+^ + sham group. ns, no significance, versus the miR-451^+/+^ + sham group. ^##^*P* < 0.01, versus the miR-451^−/−^ + sham group. ^&^*P* < 0.05, versus the miR-451^+/+^ + ICH group, *n* = 4. **D**–**F** Relative expression levels of p-ERK1/2, ERK1/2, p-AKT, and AKT protein in the brain tissues of mice in each group were detected by western blot. ^**^P < 0.01, versus the sham group. ns, no significance, versus the ICH + vehicle group. ^##^*P* < 0.01, versus the ICH + DMSO group, *n* = 4. Data are presented as means ± SD. One-way ANOVA was used to process the data, and Turkey test was performed

Previous studies indicated that MIF-dependent angiogenesis was triggered by activation of the MAPK/ERK and PI3Ks/AKT pathways. We investigated whether miR-451 regulated these pathways by targeting MIF, through applying western blot to assess the ERK and AKT phosphorylation in perihematoma region. The result indicated that the levels of p-ERK/ERK and p-AKT/AKT in perihematoma tissue of both miR-451^−/−^ and miR-451^+/+^ ICH mice were higher than that of sham group (*P* < 0.01; Fig. 6A–C). Moreover, miR-451^−/−^ mice showed considerably higher levels of p-ERK/ERK and p-AKT/AKT than those in miR-451^+/+^ mice after ICH (1.46-fold and 1.42-fold respectively; *P* < 0.01; Fig. 6A–C).

Next, MIF in miR-451^−/−^ ICH mice was inhibited by intraperitoneal injection of ISO-1 (35 mg/kg, 2 times a day, for 5 days). The p-ERK/ERK and p-AKT/AKT proteins decreased to 36.72% and 44.97%, respectively, by MIF knocking down (*P* < 0.01; Fig. 6D–F).

### Downregulation/Upregulation of miR-451 Affected Neural Functional Recovery of ICH Mouse Model

**Fig. 7:**
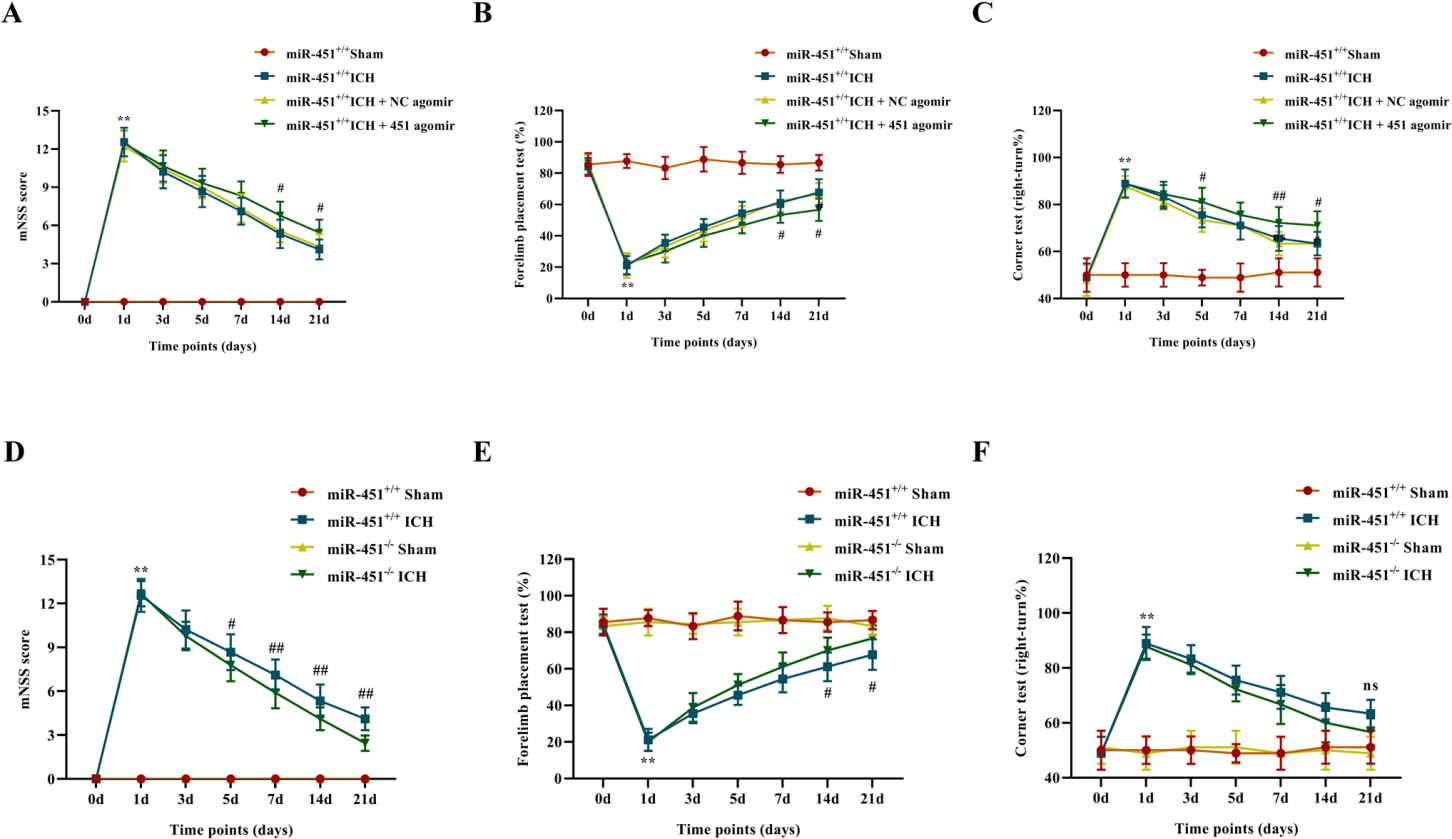
MiR-451 influenced the neural functional recovery of the ICH mouse model. **A**, **D** Modified neurological severity score (mNSS) was performed on days 0, 1, 3, 5, 7, 14, and 21 after ICH modeling or sham operation. **B**, **E** Corner test was performed on days 0, 1, 3, 5, 7, 14, and 21 after ICH modeling or sham operation. **C**, **F** Forelimb placing test was performed on days 0, 1, 3, 5, 7, 14, and 21 after ICH modeling or sham operation. ^**^P < 0.01, versus the sham group. ^#^*P* < 0.05, miR-451^−/−^ + ICH versus miR-451^+/+^ + ICH. ^##^*P* < 0.01, miR-451^−/−^ + ICH versus miR-451^+/+^ + ICH. ns, no significance, miR-451^−/−^ + ICH versus 451^+/+^ + ICH, *n* = 9. Data are presented as means ± SD. Two-way ANOVA was used to process the data, and Bonferroni test was performed

Finally, we detected whether the expression level of miR-451 affects the neural functional recovery of ICH mouse model. Modified neurological severity score (mNSS), corner test, and forelimb placing test were carried out to assess the neural function of mice at days 0, 1, 3, 5, 7, 14, and 21 after ICH modeling or sham operation. It is showed that upregulation of miR-451 was detrimental to neural functional recovery of ICH mouse model (*P* < 0.01 or *P* < 0.05; Fig. 7A–C) and downregulation of miR-451 promoted neural functional recovery of ICH mouse model (*P* < 0.01 or *P* < 0.05; Fig. 7D–E).

## Discussion

In recent years, many studies have confirmed that miRNAs are linked with a range of biological processes, such as embryonic development, hematopoietic disease, cell differentiation, proliferation, and apoptosis [30–32]. And a large number of miRNAs played an important role in angiogenesis [33, 34]. miR-134 was a tumor suppressor by targeting VEGFA (vascular endothelial growth factor)/VEGFR1 (vascular endothelial growth factor receptor 2) signaling to attenuate the progression and angiogenesis in osteosarcoma [35]. Downregulation of miR-145 in adipose-derived stem cells could promote its differentiation toward endothelial cells through the expression of ETS1 (V-ets avian erythroblastosis virus E26 oncogene homolog 1) in ischemic stroke [36]. Endothelial cell-selective deletion of the miR-15a/16–1 cluster could upregulate the protein expression of pro-angiogenic factors VEGFA and FGF2 (fibroblast growth factor 2), and their receptors VEGFR2 and FGFR1 (fibroblast growth factor receptor 1) after ischemic stroke were negative regulators for long-term neurological recovery [37]. MiR-140-5p inhibited the breast cancer invasion and angiogenesis both in vitro and in vivo by targeting VEGF-A [38]. In this study, we found that the expression of miR-451 was significantly decreased in the peripheral blood of ICH patients and decreased gradually during the first seven days. Analysis of the information of ICH patients and healthy controls revealed that the blood pressure (BP) of ICH patients, including systolic and diastolic blood pressure, was higher than that of healthy patients, and the remaining information was not significantly different. It is common sense that hypertension is a firmly established risk factor for ICH and elevated BP is common and associated with poor outcomes after ICH [39]. This was also verified by our study. It has been found that miR-451 played a role in erythrocyte development [40, 41]. So erythrocyte cleavage could release miR-451 and affected the levels of miR-451 [42]. In view of this, we have eliminated the hematoma part in the follow-up experiment process of sampling, excluding the effect of erythrocyte cleavage and release. MiR-451 continued to be decreased after ICH and remained at a low level in the acute stage until the recovery period in mouse model and in vivo model. Its expression pattern was consistent with the period of angiogenesis in previous studies, suggesting that it may play a role in regulating angiogenesis after ICH, which may be closely related to the recovery of long-term neurological function. Based on the above hypothesis, this study carried out further exploration.

Angiogenesis is a natural defense mechanism for neurological recovery after ICH, although the number of related researches in ICH is less than that after ischemic stroke. Hematologic compression of the vasculature could cause ischemia and hypoxia of local brain tissue, which then stimulated angiogenesis. In addition, the vasoactive substances released by hematoma, such as hemin and thrombin, could also stimulate vascular endothelial cells and caused endothelial cell proliferation and migration, thus forming new blood vessels. The new vascular network had strong permeability, which can significantly improve the blood supply of perihematomal region, and take away the metabolites of the hematoma to promote the absorption of the hematoma. Stamova et al. did a functional analysis of the 489 ICH differentially expressed transcripts between the ICH and control group, and found that 36 genes were involved in angiogenesis and/or vasculogenesis [43]. Tang et al. found that new vessels appeared around the hematoma and extended into it from the 7th day after collagenase-induced ICH [44]. This process depended on the function of vascular endothelial cells and was controlled by a variety of angiogenic stimulants and inhibitors. Thrombin mediates mitogenesis and survival in endothelial cells and induces angiogenesis [45, 46]. Circulating endothelial progenitor cells participate in re-endothelization and vasculogenic processes following ICH damage and favor a more extensive neurorepair [47]. Leptin triggers post-ICH angiogenesis through pericyte which is an important component of forming new blood vessels [48]. Based on the expression pattern of miR-451 was altered after ICH, we hypothesized that mir-451 could act as a factor affecting angiogenesis after ICH. The hematoma contains large amounts of hemoglobin, which was an essential for secondary damage to neurons and vascular endothelial cells. Hemoglobin could degrade into oxygenated hemoglobin, deoxygenated hemoglobin, etc., eventually releasing hemin and divalent iron ions [49, 50]. Studies have shown that hemin could accumulate in cells, causing oxidative damage to cell membrane structures, resulting in mitochondrial dysfunction and affecting cellular energy metabolism. Therefore, we chose hemin to construct an in vitro microenvironment and explored changes in cellular function after ICH. It showed that the expression of miR-451 in hBMECs decreased after hemin stimulation. Upregulation of miR-451 could inhibit the proliferation, migration, and angiogenesis of hBMECs. However, downregulation of miR-451 level could promote the function of hBMECs. We also examined the influence of miR-451 on in vitro angiogenic after ICH. We found that angiogenesis occurred in the perihematomal region in mouse model, and the angiogenesis ability was inhibited after upregulation of miR-451 level by agomir. The miR-451 knockout mice and wild-type mice were further modeled, and the detection showed that the new blood vessels increased significantly after the miR-451 knockout. It is known that BBB is formed by the cerebral endothelial cells and their linking tight junctions [51]. The mRNA expression of tight junction protein after ICH was detected to reflect the degree of BBB dysfunction, and further verified that knockdown of miR-451 could recovered tight junction and protect the integrity of BBB after ICH.

According to the results of our study, miR-451 had a role in regulating angiogenesis in both in vivo and in vitro environments, but the mechanism of its action was not clear yet and needed to be further studied. We then used target prediction tools, including TargetScan, miRanda, miRmap, and miRDB to identify that MIF was the candidate target of miR-451. The dual-luciferase reporter assays further confirmed the complementarity between miR-451 and human/mouse MIF mRNA. Upregulation/knockout of miR-451 had no effect on MIF mRNA level but could inhibit/increase MIF protein expression. Therefore, the data supported that miR-451 could inhibit MIF protein translation through binding to the 3′UTR of the mRNA rather than its mRNA transcription. MIF was an evolutionarily-conserved protein that abundantly expressed in humans and non-primate mammals which consisted of 114 amino acids and had a molecular weight of 12.5 kDa. In addition to its functions as cytokine/chemokine and angiogenic factor, the expression of MIF was strongly upregulated in several disease [52–54]. Previous studies revealed that MIF mediates endothelial cell migration and tube formation via Akt and ERK signal pathways, and could induce angiogenesis [55, 56]. We carried out further verification on this basis in ICH model. We found that the expression of MIF in miR-451 knockout mice after ICH was higher than that in wild-type mice, and the phosphorylation levels of ERK1/2 and AKT were also higher than that in wild-type mice. After administration of MIF inhibitor, the phosphorylation levels of ERK1/2 and AKT decreased, and partially offset the pro-angiogenic effect of MIF. The above results explained the mechanism by miR-451 in ICH.

MiR-451 had certain feasibility as an intervention target after ICH. The therapeutic potentials of miRNAs are gaining increasing attention, but how to use them safely and effectively still needs a lot of work to verify [57]. Zhang et al. suggested a strategy for the exosome-mediated targeted delivery of targeted delivery of miR-210 to ischemic brain and provided an effective system that can cross the BBB (blood–brain barrier) [58]. Esteves et al. used human umbilical cord blood-derived mononuclear cells as a biological vehicle to deliver miR-124-3p and evaluated its therapeutic effects in a mouse model of Parkinson’s disease [59]. The above provided the basis for the clinical application of miRNA-based therapy in the future.

However, there are still some limitations. The MIF- ERK1/2 and AKT pathway is just one role of the miR-451 signaling mechanisms that impact angiogenesis after ICH. Likewise, MIF can be regulated by various upstream factors. MiR-451/MIF pathway is not a single regulatory pathway, but part of the regulatory network after ICH. On the other hand, there are many downstream target proteins of miR-451. Whether there are other effects of miR-451 on neurological recovery after ICH is unknown. In the future, more unidentified signaling pathways of regulation after ICH need to be explored.

## Conclusions

The present study suggests that miR-451 was downregulated after ICH in the acute stage, which could promote angiogenesis directly by targeting MIF via ERK1/2 and AKT pathways after ICH. This study can provide a new possibility for modulating miRNAs to improve the prognosis of patients with ICH.

## Supplementary Information

Below is the link to the electronic supplementary material.

Supplementary file1 (DOCX 21 KB)

## Data Availability

The datasets used and/or analyzed during the current study are available from the corresponding author on reasonable request.

## Abbreviations

ICH: Intracerebral hemorrhage
miR-451: MicroRNA-451
hBMECs: Human brain microvascular endothelial cells
MIF: Macrophage migration inhibitory factor
CT: Computed tomography
BP: Blood pressure
NIHSS: National Institute of Health Stroke Scale
GCS: Glasgow Coma Scale
BBB: Blood-brain barrier
