# Supplementary material for: MicroRNA-451 Regulates Angiogenesis in Intracerebral Hemorrhage by Targeting Macrophage Migration Inhibitory Factor
Source: Mol Neurobiol. 2024 May 14;61(12):10481–99. doi: 10.1007/s12035-024-04207-3 (PMC11584486; doi:10.1007/s12035-024-04207-3)
Supplement: Supplementary file 1 — Supplementary file1 (DOCX 21 KB) [file 12035_2024_4207_MOESM1_ESM.docx]

**Supplementary Table 1.** miR-451 mimic, inhibitor and agomir sequences used in this Work

| **Gene** |  | **Sequences** | **Notes** |
| --- | --- | --- | --- |
| **miR-451 mimic** | Sense | 5’-AAACCGUUACCAUUACUGAGUU-3’ | 1:1 |
|  | Anti-sense | 5’-AACUCAGUAAUGGUAACGGU-3’ |  |
| **miR-451 inhibitor** |  | 5’-AACUCAGUAAUGGUAACGGU-3’ |  |
| **miR-451 agomir** | Sense | 5’-AAACCGUUACCAUUACUGAGUU-3’ | 1:1  with modified mature miRNA strand: 2’ OMe+5’ chol-modofied |
|  | Anti-sense | 5’-AACUCAGUAAUGGUAACGGU-3’ |  |
| **NC mimic** | Sense | 5’-UUUGUACUACACAAAAGUACUG-3’ | 1:1 |
|  | Anti-sense | 5’-CAGUACUUUUGUGUAGUACAAA-3’ |  |
| **NC inhibitor** |  | 5’-CAGUACUUUUGUGUAGUACAAA-3’ |  |
| **NC agomir** | Sense | 5’-UUUGUACUACACAAAAGUACUG-3’ | 1:1  with modified mature miRNA strand: 2’ OMe+5’ chol-modofied |
|  | Anti-sense | 5’-CAGUACUUUUGUGUAGUACAAA-3’ |  |

**Supplementary Table 2.** General patient information

| No. | Age | Sex | BP(mmHg) | | Height(m) | Weight(kg) | BMI(kg/m2) | Medical History | Volume of hematoma | Volume of edema | NIHSS | | | GCS | | |
| --- | --- | --- | --- | --- | --- | --- | --- | --- | --- | --- | --- | --- | --- | --- | --- | --- |
|  |  |  | SBP | DBP |  |  |  |  |  |  | 1d | 3d | 7d | 1d | 3d | 7d |
| ICH 1 | 70 | Female | 235 | 87 | 1.55 | 60.0 | 25.0 | HBP, Cataract | 1.5 | 1 | 3 | 3 | 2 | 15 | 15 | 15 |
| ICH 2 | 63 | Female | 168 | 87 | 1.57 | 65.0 | 26.4 | HBP | 9 | 2 | 9 | 8 | 5 | 14 | 15 | 15 |
| ICH 3 | 68 | Male | 150 | 74 | 1.70 | 50.0 | 17.3 | Autoimmune hepatitis | 10 | 3.5 | 11 | 10 | 3 | 14 | 15 | 15 |
| ICH 4 | 58 | Female | 153 | 89 | 1.60 | 70.0 | 27.3 | / | 9 | 2 | 8 | 5 | 5 | 15 | 15 | 15 |
| ICH 5 | 67 | Male | 192 | 98 | 1.64 | 64.0 | 23.8 | HBP | 1 | 2 | 3 | 3 | 3 | 15 | 15 | 15 |
| Control 1 | 61 | Female | 129 | 65 | 1.47 | 51.0 | 23.8 | HBP |  | | | | | | | |
| Control 2 | 60 | Male | 126 | 81 | 1.76 | 74.5 | 24.1 | HBP |  |  |  |  |  |  |  |  |
| Control 3 | 73 | Female | 139 | 68 | 1.57 | 65.7 | 26.8 | HBP, Diabetes, Coronary heart disease |  |  |  |  |  |  |  |  |
| Control 4 | 70 | Male | 134 | 86 | 1.72 | 70.5 | 24.0 | / |  |  |  |  |  |  |  |  |
| Control 5 | 66 | Female | 110 | 65 | 1.57 | 56.4 | 22.9 | Hysterectomy |  |  |  |  |  |  |  |  |

BP: blood pressure

HBP: high blood pressure

SBP: systolic blood pressure

DBP: diastolic blood pressure

BMI: Body Mass Index

NIHSS: National Institute of Health stroke scale

GCS: Glasgow Coma Scale
